# Recurring acquisition of carbapenemase genes and global emergence of *Pseudomonas aeruginosa* ST-1047, a lineage shaped by geopolitical conflicts

**DOI:** 10.1128/mbio.02020-25

**Published:** 2025-10-08

**Authors:** Ting L. Luo, Brendan T. Jones, Henry Dao, Viacheslav Kondratiuk, Valentyn Kovalchuk, Nadiia Fomina, Frieder Fuchs, Denis K. Byarugaba, Fred Wabwire-Mangeni, Hannah Kibuuka, Jason R. Smedberg, Ana C. Ong, Yoon I. Kwak, Antoni P. A. Hendrickx, Jason W. Bennett, Francois Lebreton, Patrick T. McGann

**Affiliations:** 1 Multidrug-Resistant Organism Repository and Surveillance Network (MRSN), Diagnostics and Countermeasures Branch, CIDR, Walter Reed Army Institute of Research 8394, Silver Spring, USA; 2 Landstuhl Regional Medical Center 25958, Landstuhl, Germany; 3 Department of Emergency and Military Medicine, National Pirogov Memorial Medical University 430804, Vinnytsia, Ukraine; 4 Department of Microbiology, National Pirogov Memorial Medical University 430804, Vinnytsia, Ukraine; 5 Department of Microbiology and Hospital Hygiene, Bundeswehr Central Hospital, Koblenz, Germany; 6 Faculty and University Hospital, Institute for Medical Microbiology, Immunology and Hygiene, University of Cologne 14309, Cologne, Germany; 7 Makerere University Walter Reed Program, Kampala, Uganda; 8 Centre for Infectious Disease Control, National Institute for Public Health and the Environment (RIVM), Bilthoven, the Netherlands; Universiteit Gent, Gent, Belgium

**Keywords:** Ukraine, antibiotic resistance, *Pseudomonas aeruginosa*, genomic epidemiology

## Abstract

*Pseudomonas aeruginosa* sequence type (ST)-1047 is emerging globally as a carbapenemase-rich lineage, yet its evolutionary history and population structure are not known. Here, we performed a comprehensive genomic and epidemiological investigation of 141 ST-1047 isolates from 15 countries, integrating short- and long-read sequencing data with Bayesian phylogenetics and mobile genetic element analyses. Two clonal subpopulations were identified. Subclone 1, defined by *bla*_VIM-11_ carriage and loss of *exoU*, is proposed to have been imported to the United States following the medical evacuation of wounded service members from Afghanistan in 2005 and later seeded a nosocomial outbreak in Washington state. Subclone 2, carrying *bla*_IMP-1_, is undergoing rapid clonal expansion due to nosocomial outbreaks in Ukraine hospitals where infection control is impaired by the war with Russia. Genomic islands resembling *P. aeruginosa* genomic island-97B mediated *bla*_IMP-1_ duplication and integration at multiple chromosomal sites, including between iron-regulated small RNAs PrrF1 and PrrF2. Outside these subclones, independent acquisitions of *bla*_NDM-1_ and/or *bla*_DIM-1_ occurred via diverse resistance islands. While plasmids were detected in some ST-1047 isolates, chromosomal integration of carbapenemase genes has promoted stability and driven the population structure. This global study reveals that, since its emergence in the late 19th century, the ST-1047 lineage showed an exceptional ability to acquire diverse carbapenemases, and that geopolitical conflicts influenced its global spread on at least two occasions. These findings underscore the need for sustained global surveillance and high-resolution genomic analyses to prevent further spread of this high-risk pathogen.

**IMPORTANCE:**

Carbapenemase-producing *Pseudomonas aeruginosa* is a major cause of healthcare-associated infections worldwide and is associated with high mortality due to limited treatment options. In this study, we characterize the emergence and international spread of a previously underrecognized lineage of *P. aeruginosa* that has independently acquired and stabilized multiple resistance genes, including those encoding VIM, IMP, NDM, and Dutch imipenemase carbapenemases. Using genomic sequencing and evolutionary analyses, we show how this lineage emerged in the late 19th century and has since adapted by integrating resistance genes directly into its chromosome, promoting long-term stability and outbreak potential. Strikingly, we link its global expansion to population movements, soldier evacuations, and healthcare disruptions during armed conflicts in Afghanistan and Ukraine. This work reveals how political instability can drive the spread of multidrug-resistant bacteria and underscores the value of high-resolution surveillance to detect and contain emerging threats before they become dominant in clinical settings.

## INTRODUCTION

*Pseudomonas aeruginosa* is an opportunistic pathogen with carbapenem-resistant strains labeled as “high” priority by the World Health Organization (1). Carbapenem-resistant *Pseudomonas aeruginosa* frequently arises from mutations reducing the outer membrane permeability and/or increasing the production of antimicrobial efflux pumps and enzymes that inactivate antimicrobials, such as the AmpC β-lactamase (2). A less common mechanism, but one that appears to be increasing in frequency (3), is attributed to the acquisition of carbapenemases via horizontal gene transfer (4).

The most frequent genes identified in carbapenemase-producing *Pseudomonas aeruginosa* (CPPA) include variants of *bla*_VIM_ (with *bla*_VIM-2_ being the most prevalent globally), *bla*_IMP_, *bla*_KPC_, and *bla*_NDM_ (3–7). These genes are frequently embedded within class 1 integrons, which are usually chromosomally located within genomic islands, although plasmid-borne carbapenemases, such as *bla*_NDM_, have also been documented (6, 7). Notably, *bla*_NDM-1_ has been found within Tn125-like transposons, facilitating its global spread in distinct CPPA lineages (7). Indeed, specific lineages are associated with carbapenemase acquisition, with sequence type (ST) ST-235 being the most dominant high-risk clone globally and has been repeatedly linked to *bla*_VIM_, *bla*_IMP_, and *bla*_GES_ genes (3). Lineages ST-111, ST-244, ST-357, and ST-308 are also frequently identified globally in association with carbapenemase genes (3–7). Rarer carbapenemases have also been detected and have been linked to regional CPPA detection with isolates carrying the Australian imipenemase, the Dutch imipenemase (DIM), or the German imipenemase, among others (7).

Unlike the high-risk global clones, *P. aeruginosa* lineage ST-1047 has only been sporadically detected, with just nine reports in the literature at the time of writing (8–16). Nevertheless, these surveys established a remarkable diversity of carbapenemases in this lineage with regional detection of *bla*_IMP_, *bla*_VIM_, *bla*_NDM_, and/or *bla*_DIM_. In particular, surveillance efforts in hospitals in Myanmar (2015–2017) and Nepal (2018–2020) revealed an early circulation in South Asia (14, 15). More recently, CPPA ST-1047 was detected throughout Europe in Switzerland (2022) and in refugees or soldiers evacuated from Ukraine to Norway (2025), Spain (2023), the Netherlands (16), or Germany (2022 and 2023), after the Russian invasion in 2022 (8–11, 13, 16).

Despite its being a hub for a variety of carbapenemases and its increasing prevalence in Europe, no comprehensive population-level analysis is available to trace the emergence and spread of lineage ST-1047. Here, we fill this knowledge gap by combining publicly available *P. aeruginosa* ST-1047 genomes to those identified from a global surveillance sampling performed by the Multidrug Resistance Organisms Repository and Surveillance Network (MRSN) through the last decade.

## MATERIALS AND METHODS

### Collections of isolates and genomes

Between 2009 and 2024, the MRSN active surveillance infrastructure sequenced 13,470 *P*. *aeruginosa* genomes in support of multiple surveillance programs scattered globally, 43 of which were ST-1047 (Table S1). Similarly, 16 ST-1047 genome sequences were obtained from surveillance efforts by the National Institute for Public Health and the Environment in the Netherlands between 2020 and 2024. Finally, all (*n* = 82) ST-1047 assemblies from the National Center for Biotechnology Information (NCBI) (as of January 2025) were included in the population analysis at the assembly level. Isolates predominantly originated from wound (23%), urine (21%), or unknown sources (19%), followed by rectal/stool/surveillance swabs (9%) and respiratory/sputum (6%) cultures. Of note, nine isolates (6%) were collected from environmental hospital surfaces in Ukraine. The full list of isolates in the study is described in Table S1.

Since 2009, the MRSN collects and analyzes clinically relevant multidrug-resistant (MDR) organisms (as defined by Magiorakos et al. [17]) across the Military Healthcare System and around the world, in collaboration with the U.S. Department of Defense Global Emerging Infections Surveillance program. Since 2020, regardless of MDR classification, the MRSN collects and sequences all *P. aeruginosa* clinical isolates across the military healthcare system. By contrast, while criteria to send isolates to the MRSN have stayed the same, the number of isolates received from global facilities is variable between years and countries and only represents a subset from submitting facilities. For example, two surveillance efforts were conducted in Ukraine: 24 *P*. *aeruginosa* spp. were collected between 2017 and 2020; none were collected in 2021; and 68 *P*. *aeruginosa* were collected between 2022 and 2024 (including 9 cultured from environmental surveillance swabs of hospital surfaces) as part of an increased surveillance effort after the invasion of Ukraine by Russia. These represent all *P. aeruginosa* spp. cultured from the war wounds of patients at these facilities during this time period.

### Whole-genome short-read sequencing

Whole-genome sequencing (WGS) was performed for all 43 isolates in possession by the MRSN. For each monoculture, genomic DNA was extracted using DNeasy UltraClean Kit (QIAGEN) and aliquoted. Library construction of genomic DNA was performed with KAPA Library Quantification Kit (Roche). WGS was performed using a MiSeq, NextSeq 500, or NextSeq 2000 Benchtop Sequencer (Illumina Inc., San Diego, CA, USA) with MiSeq Reagent Kit v.3 (600 cycles, 2 × 300 bp), NextSeq Reagent Kit 500/550 v.2 (300 cycles, 2 × 150 bp), or NextSeq 1000/2000 P2 Reagents v.3 kit (300 cycles, Illumina). Barcodes and adaptors were removed using bbduk v.38.96 (18), and reads were quality trimmed using the following parameters: ktrim = “r,” k = “23,” mink = “11,” hdist = “1,” qtrim = “r,” trimq = “15,” and minlen = “100.” Kraken2 v.2.1.2 (19) was used for initial taxonomic assignment (top hit = “1,” undetermined reads = “<10%”) and to screen for contamination (2+ genus level hits >5% = “isContaminated”). *De novo* draft genome assemblies were produced using Shovill v.1.1.0 (20), with coverage estimates generated using bbmap v.38.96 (18). Minimum thresholds for contig size and coverage were set at 200 bp and 49.5X+, respectively. Quality controls for the assembly were standardized with a decision tree including the following parameters: total length of contigs >1 Mb; total length of contigs <1 Mb over expected genome size for taxon; average read depth for each contig ≥20; total length of contigs filtered for low coverage <100 kb; total length of contigs filtered for length <100 kb; and number of contigs filtered for length + numbers of contigs filtered for low coverage <1,000. In cases where the Kraken2-derived taxonomic assignment was ambiguous, GTDB (21) taxonomic classification was used via the GTDB-Tk v.2.4.0 using a >95% average nucleotide identity threshold for species-level identification. CPPA ST-1047 of the Netherlands was sequenced as previously described (22).

### Whole-genome long-read sequencing

Within available strains at the MRSN, 10 isolates representing the genomic diversity (i.e., 10 isolates with distinct patterns of antimicrobial resistance genes and average pairwise single-nucleotide polymorphism (SNP) counts of 63.1 ± 31 SNPs, comparable to the diversity of the whole population at 60.9 ± 32 SNPs) of ST-1047 were selected for long-read sequencing on a Minion platform using a MinION Mk1B device (Oxford Nanopore Technologies, Oxford, England). The same genomic DNA used in the Illumina protocol was used for the MinION protocol. The library was prepared using the rapid barcoding kit (SQK-RBK114.96) and sequenced on an R10.4.1 flow cell for 48 hours. The resulting POD5 output was basecalled with Dorado v.9.0.0 (23) using the super-accurate model dna_r10.4.1_e8.2_400bps_sup@v.5.0.0. *De novo* assembly from long reads only was performed using Autocycler v.0.1.2 (24). Briefly, Autocycler outputs a consensus assembly from multiple long-read dedicated assemblers. The dedicated assemblers used were Flye, Raven, Miniasm, Metamdbg, Necat, NextDenovo, and Redbean.

### Molecular typing and annotations

Genomes were annotated using Bakta v.1.10.4 (25). Antimicrobial resistance genes were annotated using a combination of ARIBA v.2.14.6 (26) and AMRFinderPlus v.4.0.19 (27). Thresholds for AMR calls include ident_min <0.9> and coverage_min <0.5> for AMRFinderPlus; nucmer_min_id <90> nucmer_min_len <20> and nucmer_breaklen <200> for ARIBA. Finally, a 10× minimum coverage was applied for ARIBA-only hits, and deduplication was performed with priority given to the assembly-based hit. Multilocus sequence typing (MLST) assignment was performed using MLST v.2.22.1 (28). Mobile genetic elements (MGEs) were identified and annotated with tools and databases such as mobileOG-db (beatrix-1.6) (29), ISFinder (30), and TnCentral (31). Plasmid replication initiation genes (Rep types) were determined using PlasmidFinder (32) for all 141 draft genomes. Visualization of MGEs was generated using BLAST that is embedded in EasyFig tool (33). Parameters included an expected value cutoff of 0.0001, filtering of low complexity regions, filtering of regions with <95% nucleotide identity, and filtering alignment lengths of less than 1,000 bp).

### Core genome MLST, SNP calling, and phylogenetic analysis

Core genome MLST was performed in SeqSphere+ Software v.7.7.2 (Ridom, Germany) using the cgMLST schemes developed for *P. aeruginosa* (34) using a cutoff of 90%. A cgMLST minimum-spanning tree of all 141 ST-1047 assemblies and 1 representative of each *P. aeruginosa* sequence type in the MRSN repository was generated to identify lineages most closely related to ST-1047.

SNP calling was performed with Snippy v.4.4.5 (35) using error correction (Pilon v.1.23) (36), and the genome of the earliest isolate (by date) was chosen as reference. The core alignment length of 7,177,982 bp was filtered for recombination using Gubbins v.2.3.156 (37), and an SNP-based phylogeny was created by inferring a maximum-likelihood tree with RaxML-NG v.0.9.0 (38) using the GTR+G model and 50-50 parsimony and random starting trees.

A dated phylogeny was constructed with BEAST2 (39) on all 83 ST-1047 samples with Illumina short-read data at an estimated depth of at least 50×. This included all 43 samples sequenced by the MRSN and 40 external samples harvested from NCBI’s Sequence Read Archive (Table S1). An outgroup of eight ST-377 *P. aeruginosa* isolates, the closest lineage to ST-1047 by cgMLST distances, was also included in the analysis. BEAST2 parameters used include the HKY substitution, random clock, and coalescent constant population models and a Markov chain Monte Carlo length of 1 × 10^8^ with sampling every 5 × 10^3^ steps. Analysis was repeated five times to ensure the posterior distributions remain consistent. A 10% burn-in was implemented to summarize all posterior trees, and a maximum clade credibility (MCC) tree was constructed in TreeAnnotator v.2.7.6. The MCC tree was visualized in iTOL (40) layered with corresponding genomic and metadata descriptors. When necessary, manual annotation and superficial edits were made using CorelDRAW.

## RESULTS

### A global collection of ST-1047 *Pseudomonas aeruginosa* isolates and genomes

The starting data set (*n* = 141) was, as of January 2025, exhaustive of all ST-1047 genomes available in the public domain (*n* = 82), as well as newly generated sequences for this study (*n* = 59) (Table S1). At the species population level, cgMLST analysis places the 141 ST-1047 isolates as a monophyletic group, within the *exoU*^+^ clade of *P. aeruginosa* (Fig. S1). The nearest neighbor from outside this lineage in the entire MRSN collection belonged to lineage ST-377, a single locus variant of ST-1047 by traditional MLST.

**Fig 1:**
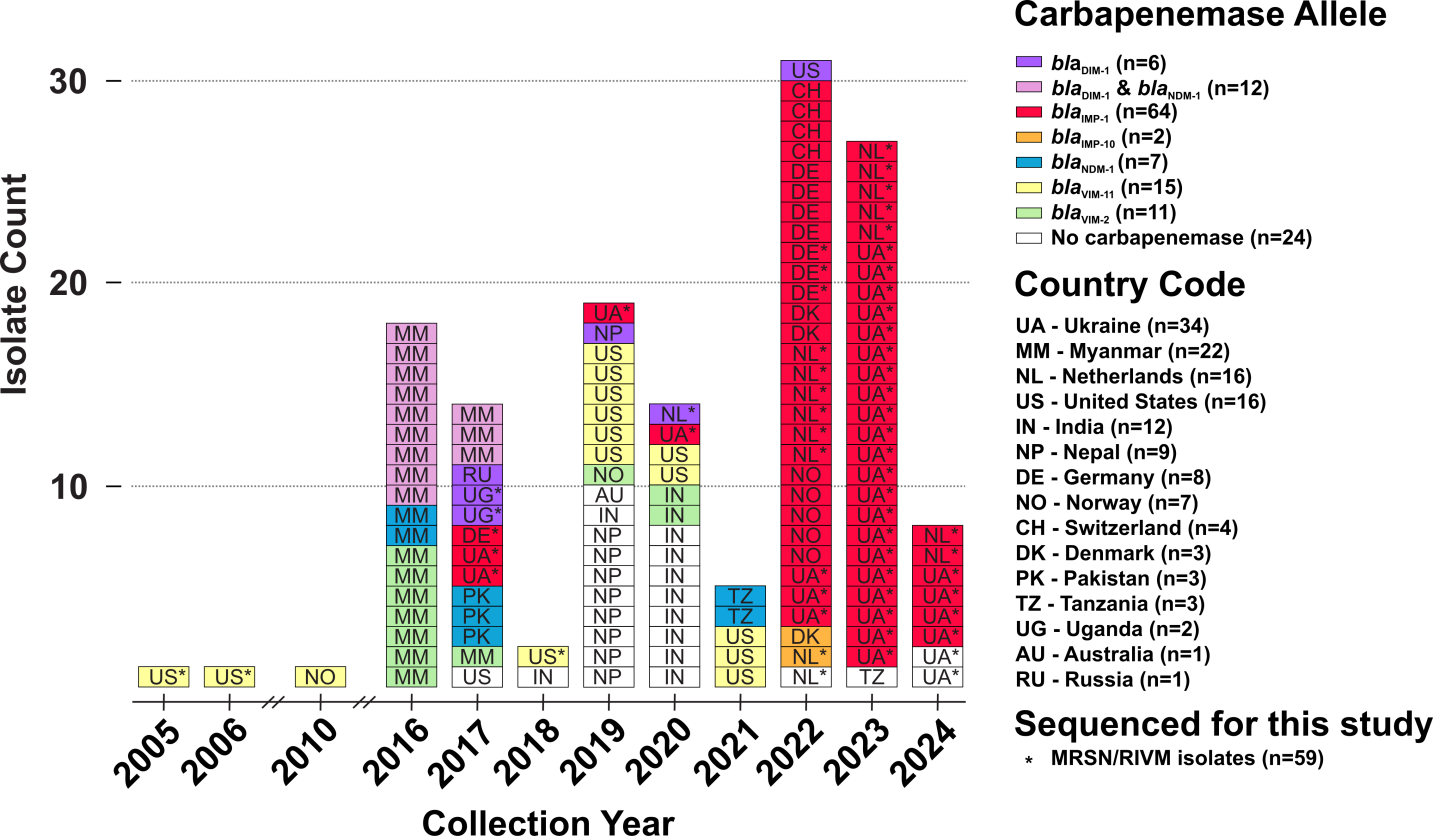
A global, comprehensive collection of 141 ST-1047 *P. aeruginosa*. Epidemic curve of all 141 ST-1047 *P. aeruginosa* reveals higher incidence between the years 2022 and 2024 and is saturated with the presence of *bla*_IMP-1_. Other carbapenemase families have been detected in the lineage, including *bla*_DIM_, *bla*_VIM_, and *bla*_NDM_. The geographical reach of ST-1047 is wide, with cases documented in countries from four continents. The countries most represented are Ukraine (*n* = 34), Myanmar (*n* = 22), the Netherlands (*n* = 16), United States (*n* = 16), and India (*n* = 12).

Geographically, the ST-1047 originated from 15 countries with Ukraine (*n* = 34), Myanmar (*n* = 22), the Netherlands (*n* = 16), the United States (*n* = 16), and India (*n* = 12) being the most represented. Temporally, the isolates were collected from 2005 to 2024, although 66 of the 141 isolates (47%) were collected in 2023 and 2024 (Fig. 1). The skew in temporal and geographical distribution is largely explained by variability in the sampling and does not necessarily reflect true prevalence. For example, of the 43 MRSN isolates/genomes, 33 (77%) were collected between 2022 and 2024 (Fig. 1). All were sourced from patients with known links to Ukraine (*n* = 24), or from environmental samples taken at Ukrainian hospitals (*n* = 9), as part of an increased surveillance effort in this country. Besides the MRSN sampling, further patterns were explained by regional surveillance efforts in Myanmar (2015–2017) (14), Nepal (2018–2020) (15), or India (2010–2020) (submitted to GenBank by Bhabatosh Das within BioProject PRJNA924087) or in a single U.S. hospital by the Washington State Department of Health (2019–2021) (submitted to GenBank by Hannah Gray within BioProject PRJNA288601).

### Carriage of IMP, VIM, NDM, or DIM carbapenemase in global ST-1047

Within this data set, 116/141 (82%) of the isolates carried at least one carbapenemase gene with variants of *bla*_IMP_ (*n* = 66, 47%), *bla*_VIM_ (*n* = 26, 18%), *bla*_NDM_ (*n* = 19, 13%), and *bla*_DIM_ (*n* = 18, 13%) being represented. The remaining 25 isolates carried no carbapenemase and were largely from India (*n* = 10) and Nepal (*n* = 8) (Fig. 1). Six isolates only carried *bla*_DIM-1_, while the remaining 12 co-carried *bla*_DIM-1_ and *bla*_NDM-1_. The former were collected from all around the globe with two isolates from Uganda and a single isolate from Russia, Nepal, Norway, and the United States. By contrast, the carriage of the other carbapenemases largely correlated with the geographical and/or temporal origins of the isolates, and three main groups were identified.

First, of the 66 isolates carrying *bla*_IMP_, 64 carried *bla*_IMP-1_, and 2 carried its single nucleotide variant *bla*_IMP-10_. Temporally, the carriage of an IMP carbapenemase was significantly higher in isolates collected after 2022 (92%) compared to earlier isolates (7%; chi-sq *P*-value < 0.001) (Fig. 1). Geographically, IMP-producing isolates were exclusively detected in Europe. Of the 34 isolates collected in Ukraine, 32 (94%) carried *bla*_IMP_, and of the 38 isolates collected in other European countries (the Netherlands, Germany, Denmark, Switzerland, and Norway), 34 (89%) carried *bla*_IMP_.

Second, of the 26 isolates carrying *bla*_VIM_, 15 carried *bla*_VIM-11_ and 11 carried its single-nucleotide variant, *bla*_VIM-2_. While all were collected prior to 2022, no strong temporal patterns were observed, and sporadic detections of VIM-carrying isolates were made throughout 2005–2021 (Fig. 1). Interestingly, in part due to the outbreak in a Washington state hospital (unpublished data), *bla*_VIM-11_ was largely enriched in U.S. isolates (67%) compared to other countries (2%, chi-square *P* value < 0.001). By contrast, *bla*_VIM-2_ was most frequently found in isolates from Myanmar (36%) and only rarely from other countries (3%, chi-square *P* value < 0.001).

Third, of the 19 isolates carrying *bla*_NDM_, all carried *bla*_NDM-1_, and a subset of 12 isolates from Myanmar also carried the *bla*_DIM-1_ carbapenemase gene (Fig. 1). No significant temporal signal was identified, but geographically, NDM-carrying isolates were largely encountered in South Asian countries (41% of isolates from Myanmar, Pakistan, India, and Nepal) compared to the rest of the world (2%, chi-square *P* value < 0.001).

Finally, other antimicrobial resistance genes were identified in ST-1047 genomes (Table S1). The most notable included the *rmtB/F* 16S methyltransferase genes detected in 22 isolates with a higher prevalence in South Asian countries (39% of isolates from Myanmar, Pakistan, India, and Nepal) compared to others (4%, *P* value < 0.001), and the rare detection of extended spectrum beta-lactamase genes *bla*_GES-9_ (*n* = 5 from India), *bla*_CTX-M-15_ (*n* = 1 from Norway), and both *bla*_CTX-M-15_ and variants of *bla*_VEB_ in six isolates from Myanmar (Table S1).

### Import and clonal expansion of VIM-carrying ST-1047 in the United States, with origins in the Middle East

**Fig 2:**
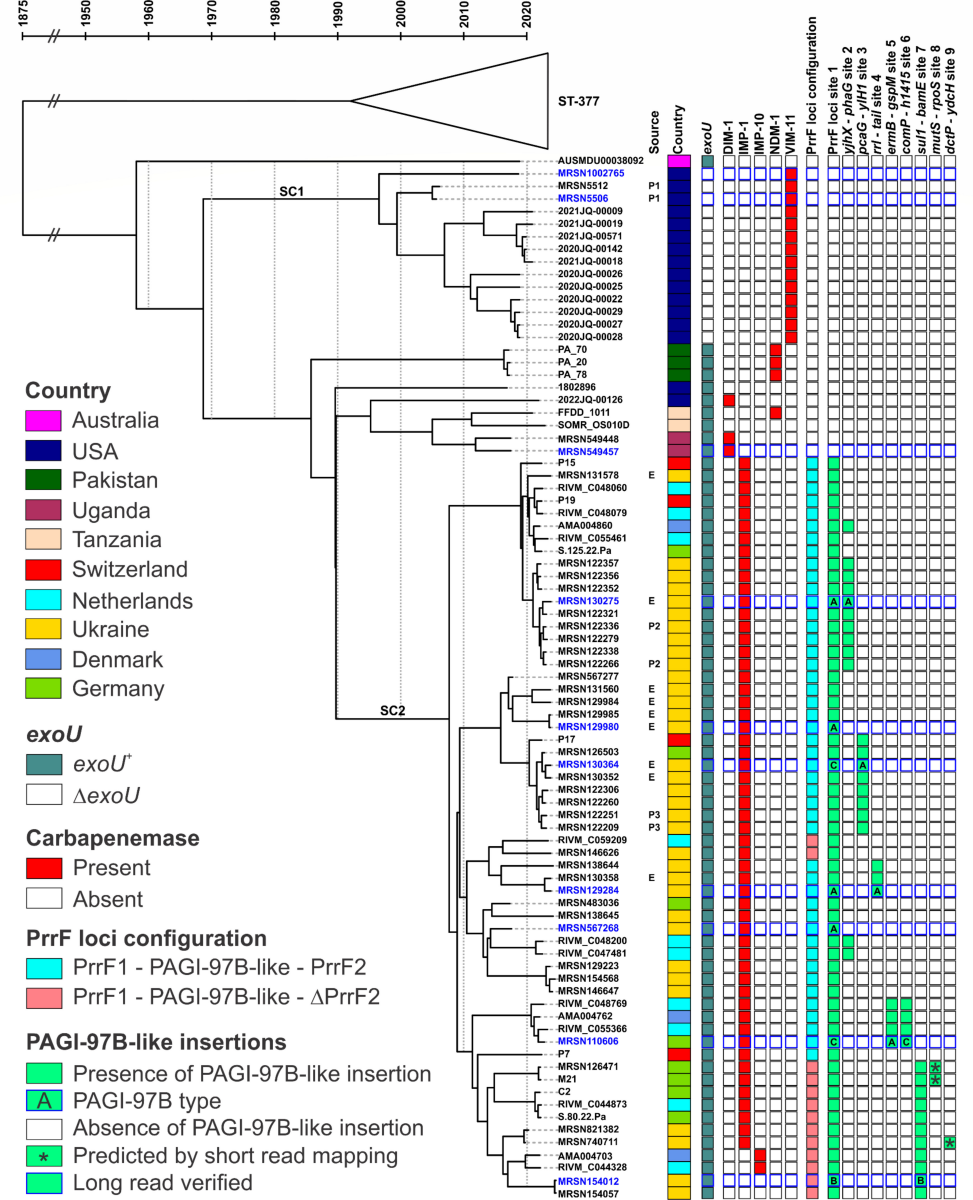
Time-stamped phylogeny reveals the population structure of ST-1047 *P. aeruginosa*. Dated BEAST2 phylogeny reveals the population structure of 83 ST-1047 and eight closely related ST-377 *P. aeruginosa*. The two lineages are separated by approximately 425 SNPs, and the MRCA of these two lineages dates back to 1875. The most prominent features of ST-1047 are a large (*n* = 59) monophyletic clade (SC2) carrying all *bla*_IMP-1/10_ isolates and a smaller monophyletic clade (SC1) of *bla*_VIM-11_-carrying isolates. Clustering by carbapenemase suggests one horizontal acquisition event followed by clonal expansion. In the SC2 clade, cataloging *P. aeruginosa* genomic island (PAGI)-97B-like insertion sites and presence/absence of PrrF2 reveals further structuring. Incremental acquisition of PAGI-97B-like genomic islands in the genome is a recent evolutionary event and coincides with the Russo-Ukrainian War. Where appropriate, environmental sources and patient serial isolates are indicated.

To reconstruct the evolution and acquisition of carbapenemases within the ST-1047 lineage, a time-calibrated phylogeny was constructed for the subset of 83 isolates for which quality read sets were available (Fig. 2). ST-1047 is predicted to have diverged from its nearest-neighbor ST-377 in the late 19th century, and the most recent common ancestor (MRCA) to our collection dated to 1958 (95% highest posterior density interval 1935–1996). Since then, two subclones (SCs) have emerged and are stratified by the carriage of *bla*_VIM_ (SC1) and *bla*_IMP_ (SC2).

Three isolates branched out at the origin of SC1 in the late 1990s (Fig. 2). One (1002765) originated from the CDC and FDA Antimicrobial Resistance Isolate Bank, but its date of collection and specific origin are unknown. The two others (5506 and 5512) were the earliest VIM-carrying isolates in the collection and were obtained from a U.S. service member wounded and stabilized in Afghanistan in 2005 and evacuated to the United States. This isolate was cultured from an arm injury immediately upon arrival in the United States, indicating it was acquired during prior hospitalization in Afghanistan. These three historical isolates are genetically distinct by only 22–37 SNPs from 11 VIM-carrying isolates collected throughout 2019–2021 during a suspected nosocomial outbreak at a U.S. civilian hospital in Washington state (genomes submitted to GenBank by Hannah Gray within BioProject PRJNA288601) (Fig. 2).

**Fig 3:**
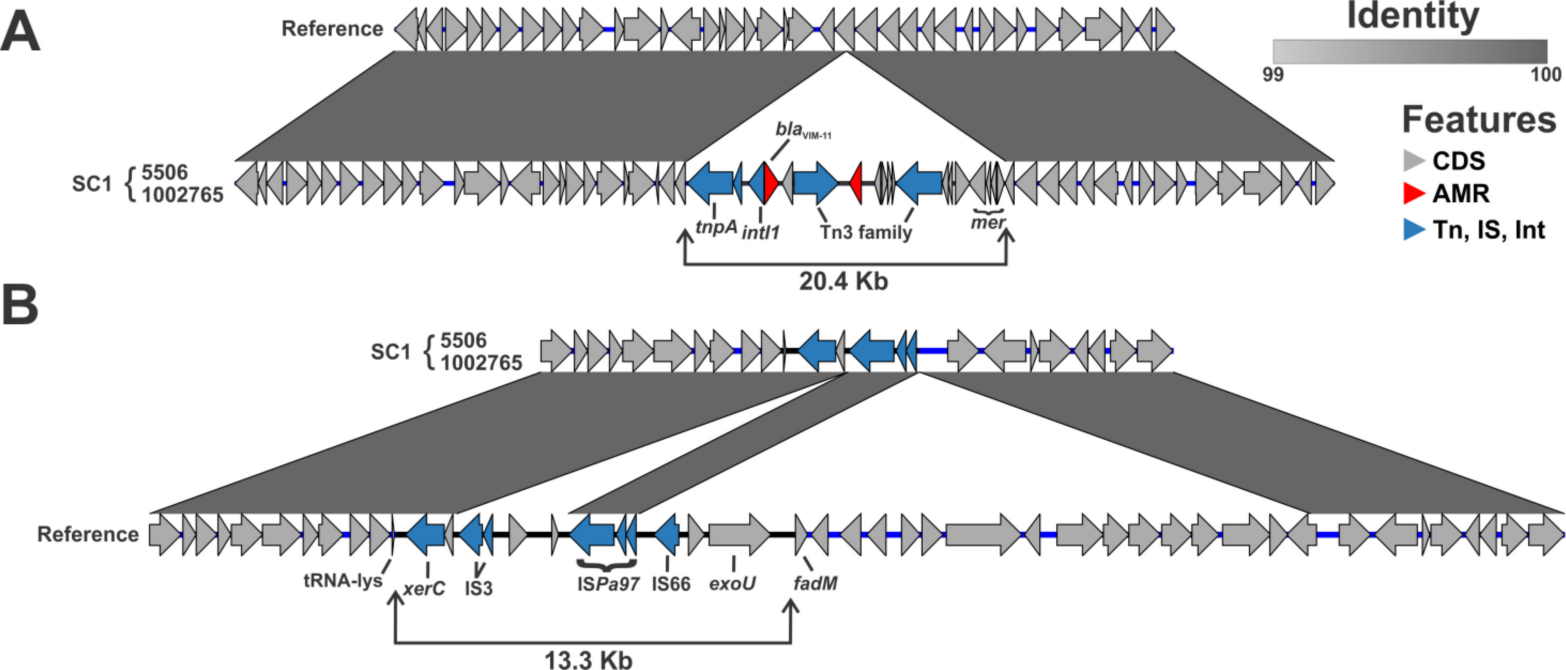
Defining features at the origins of the VIM-producing ST-1047 subclone 1. Pairwise alignment shows the acquisition of *bla*_VIM-11_ involved a 20.4 kb transposon (**A**). MRSN5506, MRSN1002765, and the reference AP017302.1. Reference AP017302.1 was used as the reference because the chromosomal region where the transposition occurred was fully intact. Pairwise alignment shows the loss of *exoU* in subclone 1 of ST-1047 *P. aeruginosa* involved two deletion events (**B**). Remnants of the genomic island carrying *exoU* in reference 549457 are evident in SC1 isolates.

From the population structure and maximum parsimony, a single acquisition event of the bla_VIM-11_ carbapenemase gene is predicted as the origin of SC1 (Fig. 2). This was confirmed by the analysis of the complete genomes for two selected isolates (1002765 and 5506), which revealed an identical site of chromosomal integration (interrupting a putative aspartate aminotransferase) by a 20.4 kb *Tn*501-like transposon that includes genes encoding a transposase (*tnpA*) and a resolvase (*tnpR*), an inserted *bla*_VIM-11_-harboring class 1 integron, and a *mer* operon (Fig. 3A). Using short-read mapping, the same insertion site was predicted to be shared by all other isolates within SC1 (not shown). Another defining feature as the origins of SC1 was noted; all isolates lacked the *exoU* gene compared to the ancestral ST-377 and all remaining ST-1047 isolates (Fig. 2). A 13,325 bp genomic island harboring *exoU* (GI-*exoU*) was detected in the reference. The boundaries are downstream of the tRNA-lys and upstream of *fadM* (Fig. 3B). Two deletion events excised roughly 8.9 kb of GI-*exoU*, leaving 4.4 kb of GI-*exoU* as remnants in SC1 isolates. These remnants confirmed the presence of the GI-*exoU* that was lost in the MRCA of SC1 (Fig. 3B).

### Recent spread in Europe of IMP-carrying ST-1047 linked to war in Ukraine

BEAST inferences date the emergence of subclone 2 between 1990 and 2008 (Fig. 2). Remarkably, apart from the four *bla*_IMP-1_-carrying isolates from Switzerland, for which no patient data were available, all other IMP-carrying SC2 isolates can be traced to Ukrainian patients, hospital surfaces in Ukraine, or Ukrainian soldiers and refugees evacuated to other countries in Europe (Table S1) (9–11, 13, 16). Besides the epidemiological data, the tree topology reveals a clonal expansion with SNP distances within the SC2 isolates ranging from 0 to 93 SNPs (Table S2).

**Fig 4:**
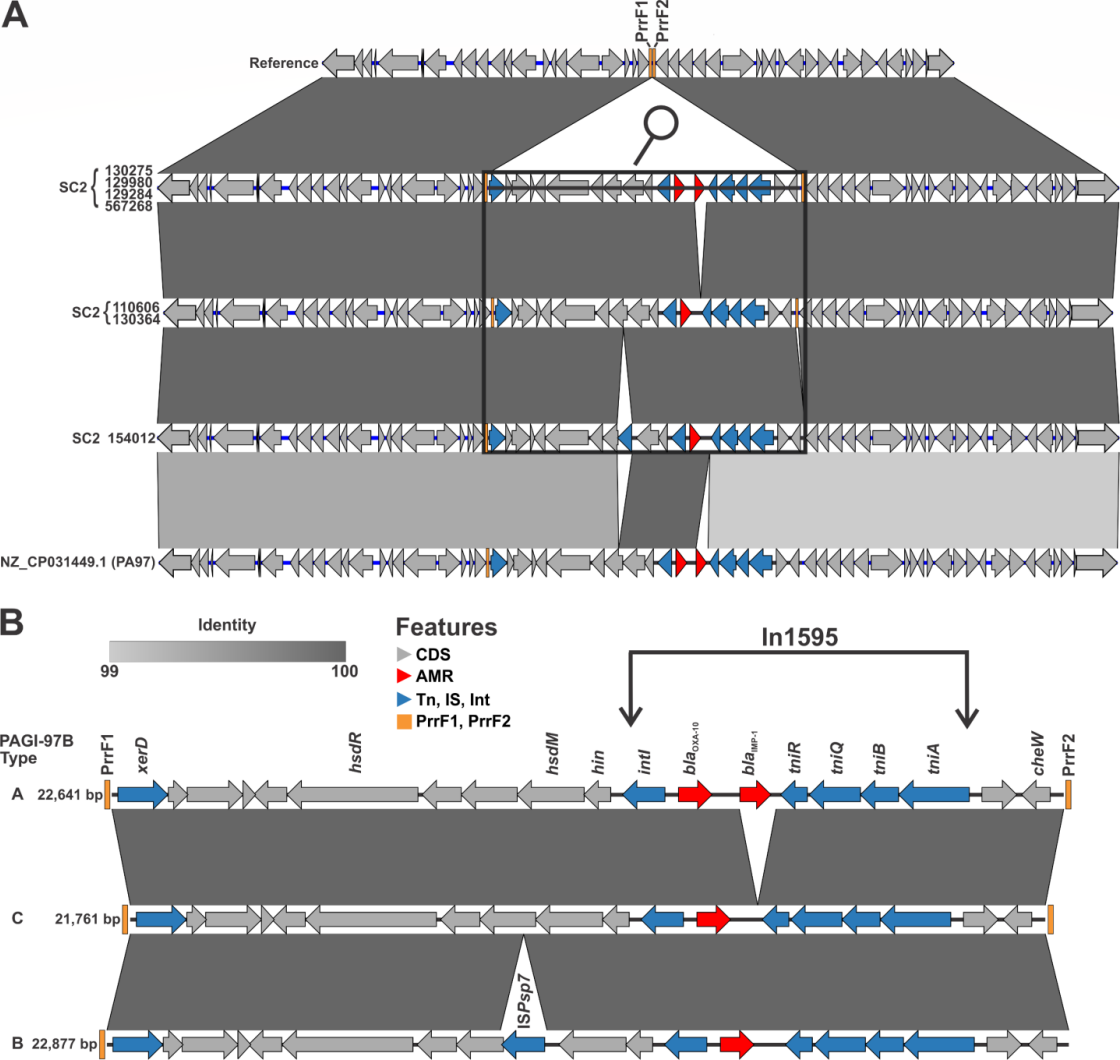
Defining features at the origins of the IMP-producing ST-1047 subclone 2. PAGI-97B 22.6 kb genomic island was acquired from one genetic event in ST-1047 *P. aeruginosa*. Pairwise alignments (**A**) of PAGI-97B-like genomic island variants show aligned regions with 100% identity and presence of indels, suggesting singular genetic events integrating and excising shorter sequences. A zoomed-in visualization (**B**) of the PAGI-97B-like variants shows that it is integrated by *xerD* recombinase with *bla*_IMP-1_ bracketed by In1595. Alignment to PA97 ST-234 chromosome, as described in Janice et al. (41), shows that PAGI-97B is identical to type A, and the transposed sequences are bracketed by ST-1047 and ST-234 backbones that are 99% identical.

Within SC2, all isolates carried *bla*_IMP-1_ except for two isolates (distinct to each other by only eight SNPs) that carried the one nucleotide variant *bla*_IMP-10_ and two genetically identical isolates (154012 and 154057) that lacked this carbapenemase gene (Fig. 2). As such, a single acquisition event of the IMP carbapenemase gene was predicted as the origin of SC2 by maximum parsimony analysis. This was confirmed by the analysis of mobile genetic elements in closed genomes of SC2 isolates compared to reference isolate 549457, the closest neighbor outside of SC2. All seven selected SC2 genomes (including 154012, which lacked *bla*_IMP_) carried a chromosomal genomic island (homologous to *P. aeruginosa* genomic island [PAGI]-97B, first identified from an extensively resistant ST-234 strain from Ghana [41]) harboring an In1595 class I integron (Fig. 4A). Besides the select closed genomes, short-read mapping confirmed that the insertion site of PAGI-97B, between the tandem small RNAs PrrF1 and PrrF2 (site 1), was conserved for all SC2 isolates. However, this also revealed that at two independent instances throughout the evolution of SC2, a 209 bp deletion led to the excision of PrrF2, just downstream of PAGI-97B (Fig. 2; Fig. S2).

Three types (labeled A–C) of PAGI-97B were found inserted at site one in the seven circularized SC2 genomes (Fig. 4A). Type A was a 22.6 kb molecule carrying *bla*_IMP-1_ (Fig. 4B). Type B, in SC2 isolate 154012 lacking a carbapenemase, was a 22.9 kb variant with *bla*_IMP-1_ excised from In1595 and an insertion of ISPsp7. Finally, type C was a 21.8 kb variant with a deletion of *bla*_IMP-1_ but without the ISPsp7 insertion (Fig. 4B). Interestingly, PAGI-97B type C was found inserted in site 1 of isolates 110606 and 130364, which did carry a duplicate *bla*_IMP-1_ carbapenemase elsewhere in their genome. Further analysis of 110606 and 130364 revealed that PAGI-97B-like elements existed in multiple copies (two and three, respectively) inserted at distinct sites in their genomes (Fig. 2). In particular, PAGI-97B type A with *bla*_IMP-1_ was found inserted between loci *pcaG-yIH1* (site 3) in isolate 130364 and between loci *ermB-gspM* (site 5) in 110606 (Fig. 2). Analysis of all SC2 isolates identified that, while all carried a PAGI-97B-like molecule in PrrF loci site 1, subsets of isolates carried either one (*n* = 31) or two (*n* = 7) additional copies inserted at variable chromosomal locations (sites 2–9) (Fig. 2).

### Recurring, independent acquisitions of *bla*_DIM-1_ or *bla*_NDM-1_ in ST-1047 from global origins

**Fig 5:**
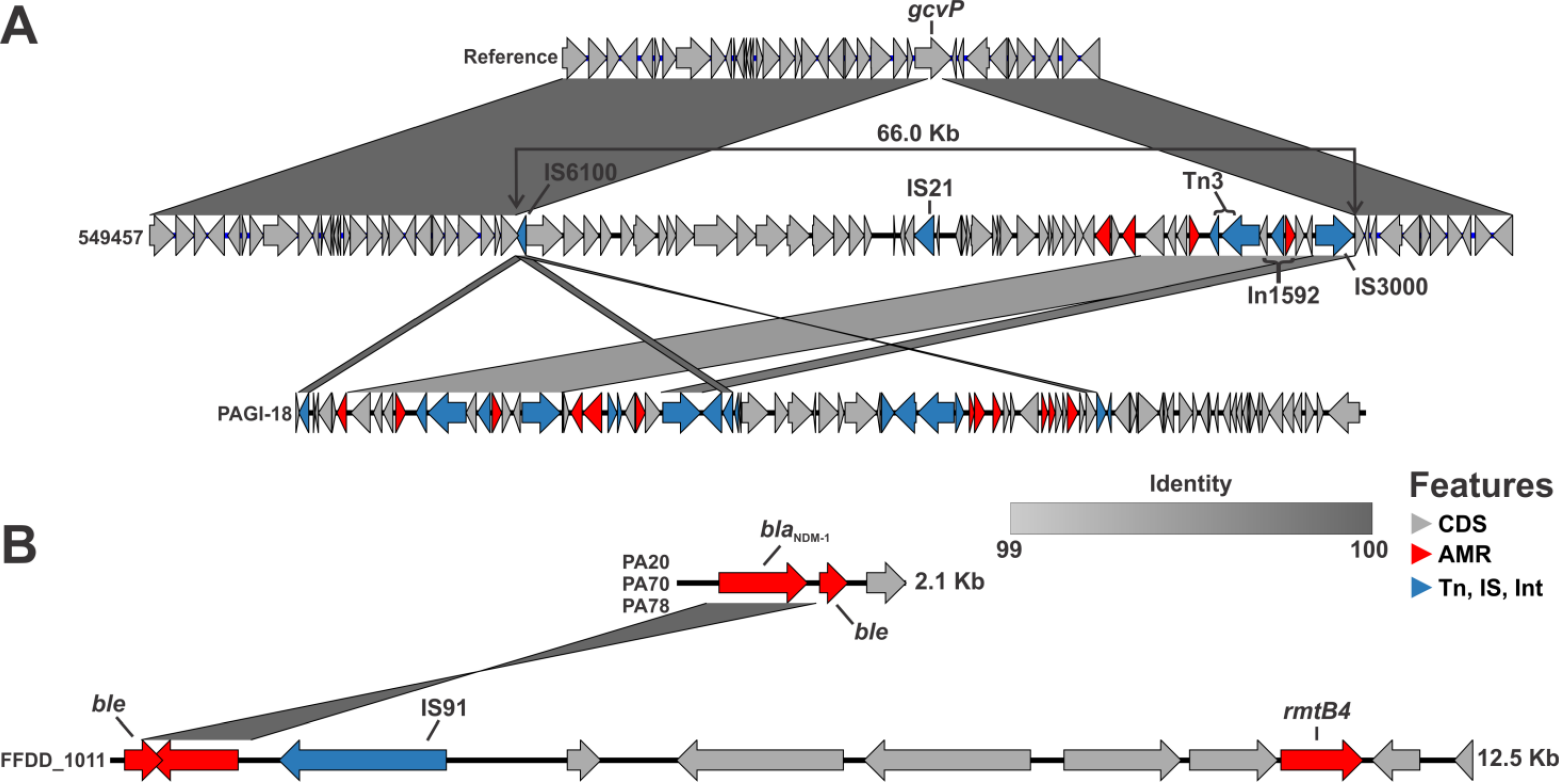
Independent, recurring acquisition of *bla*_DIM-1_ and *bla*_NDM-1_ in ST-1047. Acquisition of *bla*_DIM-1_ involved a 66 kb genomic island that was inserted into *gcvP* in the chromosome (**A**). This genomic island shares 16.6 kb of uninterrupted homology at over 99% identity with PAGI-18 and includes In1592 carrying *bla*_DIM-1_. Both genomic islands are flanked by IS6100 at the 5′ end. Full-length contigs containing *bla*_NDM-1_ in isolates PA20/70/78 were compared to full-length contigs containing *bla*_NDM-1_ in FFDD_1011 (**B**). No homology was detected outside the *bla*_NDM-1_ sequence, suggesting separate mobile elements were independently acquired by ST-1047 *P. aeruginosa*.

Outside of SC1 and SC2, the remaining isolates carrying either *bla*_DIM-1_ or *bla*_NDM-1_ did not form single monophyletic groups, suggestive of independent acquisition events (Fig. 2). Three isolates carried *bla*_DIM-1_: 549457 and 549448, two highly genetically related isolates from Uganda (distinct by 16 SNPs), and 2022JQ-00126, which was collected from the United States and only distantly genetically related (Fig. 2). Analysis of the closed genome for 549457 revealed that *bla*_DIM-1_ was part of type I integron In1592 (first identified in an extensively resistant ST-234 strain from Ghana [41]). However, the integron was itself carried by a 66 kb genomic island which shared a more significant homology with PAGI-18 (Fig. 5A), detected in a *P. aeruginosa* ST-234 from Poland (42). Both genomic islands are flanked by IS6100 at the 5′ end and share 16.6 kb of uninterrupted sequence at over 99% homology. In 549457 and 549448 (verified by short-read mapping), the genomic island was chromosomally inserted within the glycine dehydrogenase gene *gcvP* (Fig. 5A). No closed genome was available for 2022JQ-00126, but the *gcvP* locus in the short-read assembly was uninterrupted and read mapping to the 66 kb genomic island revealed gaps (not shown). This confirmed *bla*_DIM-1_ in 2022JQ-00126 was independently acquired, carried by a similar genomic island to 549457, and inserted elsewhere in the genome.

 Finally, four isolates carried *bla*_NDM-1_: three genetically identical isolates from Pakistan (PA20, PA70, and PA78) and isolate FFDD_1011 from Tanzania (Fig. 2). No closed genomes were available to investigate the mobile genetic elements harboring *bla*_NDM-1_ in these isolates. Nevertheless, contextual clues can be gleaned from the short-read contigs that carry *bla*_NDM-1_. These contigs are likely flanked by ISs or repeat regions that break contigs in short-read assemblies. The isolates from Pakistan had *bla*_NDM-1_ carried on identical short 2.1 kb contigs (Fig. 5B), suggesting that *bla*_NDM-1_ is carried on an element with a high density of ISs. Contrastingly, the isolate from Tanzania had IS91 upstream of *bla*_NDM-1_, reminiscent of the IS91-*bla*_NDM_-1-IS91 configuration observed in the ST-1047 from Myanmar (14). The contig also carried *rmtB4* a few coding sequences upstream of *bla*_NDM-1_, indicating that the carbapenemase is carried on a different mobile element than the *bla*_NDM-1_ from Pakistani isolates (Fig. 5B). Additionally, all Pakistani isolates were lacking *rmtB4*. While the insertion site could not be identified, the boundaries of the *bla*_NDM-1_ carrying contigs were distinct between FFDD_1011 and PA20/PA70/PA78, supporting independent acquisition events.

## DISCUSSION

To date, this study is the first global and comprehensive genomic epidemiology and molecular analysis of ST-1047 *P. aeruginosa*. This lineage warranted special attention for two reasons: its propensity to acquire various carbapenemases, including the rarer *bla*_NDM_ and *bla*_DIM_ (14), and its recent rapid emergence throughout Europe, associated with the war in Ukraine. Geopolitical conflicts have long been known to facilitate the spread of MDR pathogens. This has been documented in the American-led operations in Iraq and Afghanistan (2003–2021), the Libyan Civil War (2014–2020), the Syrian Civil War (2011–present), and the ongoing Russo-Ukrainian conflict (9, 11, 13, 19, 41–47). Here, we propose that, twice, armed conflicts have influenced the global spread of ST-1047 *P. aeruginosa*.

First and most recently, our data reveal a rapid clonal expansion of IMP-producing subclone 2, with origins tied to Ukraine. With a MRCA dated between 1990 and 2008, we confirm our recent report that subclone 2 pre-existed in Ukraine prior to the Russian invasion in 2022, and that the clonal expansion results from nosocomial outbreaks caused by disruptions in infection control (48). Since 2022, refugees, evacuated wounded soldiers, or repatriated foreign fighters have facilitated the spread of IMP-producing ST-1047 from Ukraine to Norway, Spain, Germany, the Netherlands, and Denmark and possibly other European countries where it has so far escaped detection (9–11, 13, 16).

Second, historically, our analysis suggests that the VIM-11 producing subclone 1, which caused an outbreak in a U.S. hospital in the State of Washington in 2019–2021 (unpublished data), was first imported in the United States by service members wounded during Operation Enduring Freedom (2001–2014) and evacuated from Afghanistan. While this inference is built upon only two isolates from a single soldier, additional observations support this hypothesis. Genetically, at the population level, subclone 1 isolates are most closely related to ST-1047 from Pakistan, suggestive of an MRCA circulating in the region. Genetically again, three factors support that subclone 1 arose from a single introduction in the United States: (i) a shared *bla*_VIM-11_ insertion site, (ii) a shared and unique *exoU* deletion, and (iii) SNP differences between the soldier and the Washington State outbreak isolates amounting to 2.64 SNPs/year, well within the expected evolution rate for isolates with direct ancestry (49, 50). Finally, epidemiologically, reviews of the soldier history revealed a known epidemiological link to Washington State, although a direct match to the hospital with the ST-1047 outbreak could not be established.

Besides the VIM- or IMP-producing subclones 1 and 2, the Bayesian phylogenetic analysis did not reveal large monophyletic clades of isolates carrying the NDM or DIM carbapenemase. In fact, for each, two distinct acquisition events were inferred. However, it should be noted that the temporal and regional heterogeneity of the strains included in the study, along with the inclusion of multiple isolates from hospital outbreaks, likely influenced the inferred population structure. Furthermore, 58 isolates (including 15 with *bla*_NDM-1_) with only draft genomes and no sequencing read data available could not be included in this high-resolution analysis. These represented all genome sequences from Myanmar, Nepal, and India (14, 15). Considering that a strong enrichment of NDM-carrying ST-1047 isolates was noted in Myanmar and Pakistan (14), a hypothesis can be made that an NDM-producing subclone is circulating in the region. High-quality genome data would be needed to investigate this and the fine population structure of ST-1047 in South Asia.

A remarkable hub for carbapenemases from four distinct families (VIM, IMP, NDM, and DIM), lineage ST-1047 was of particular interest to study the mobile genetic elements and the route of emergence of CPPA isolates. Remarkably, all carbapenemase genes found in this lineage were chromosomally integrated, except for *bla*_NDM-1_ in Pakistan and Tanzania isolates, for which no determination could be made (no long-read sequencing data available). In ST-1047, *bla*_VIM-11_ (within a novel type I integron on a *Tn*501-like transposon), *bla*_IMP-1_ (In1595, carried by PAGI-97B-like island [41]), and *bla*_DIM-1_ (In1592, carried by a PAGI-18-like island [42]) were carried by so-called *P. aeruginosa* genomic islands (PAGIs) (7).

PAGIs are the hallmark of high-risk CPPA clones and, in the most recent comprehensive inventory, over 45 diverse resistance islands carrying carbapenemase genes were categorized by Yoon and Jeong (7). Here, we note that PAGIs influenced the evolution of ST-1047 in three ways: (i) a higher stability of the carbapenemase genes, a known feature (51) of chromosomal insertions, which explains the distinct population structure of this lineage; (ii) a stable bank of resistance genes from which duplications can be made (e.g., up to three copies of the *bla*_IMP-1_ genomic island observed within SC2) possibly to increase resistance levels (14, 52) or the likelihood of horizontal dissemination (53, 54); and (iii) an adaptive strategy through their insertion and disruption of chromosomal genes, operons, or sRNA.

It is documented that transposition of genomic islands into the chromosome can disrupt genetic features at the site of integration and affect the physiology and pathogenicity of the recipient (54–56). Within all insertion sites of carbapenemase-carrying PAGIs we identified in ST-1047, one was of particular interest. In subclone 2, the primary insertion of IMP-carrying PAGI-97B-like was mapped precisely between the tandem small RNAs PrrF1/PrrF2. This site has been previously identified as a hotspot for chromosomal integration of *xerD*-encoding PAGIs (41). PrrF1 and PrrF2 are two highly identical (95%) small regulatory RNAs whose expression is induced under iron limitation and is required for virulence in acute murine lung infection models (57, 58). Of particular interest, after the PAGI-97B-like insertion, the precise excision of PrrF2 was achieved at two independent occasions throughout the evolution of SC2. Whether the acquisition of PAGI-97B-like or the subsequent deletion of PrrF2 impacts the virulence of ST-1047 subclone 1 remains to be determined. In subclone 1, although independent from the acquisition of its IMP-carrying PAGI, the deletion of *exoU*, which has been proposed as a mechanism for *P. aeruginosa* to evade host immune factors (59), is another indication suggesting that lineage ST-1047 may be adapting to the human host and evolving to better balance virulence and resistance.

To conclude, our global study retraced the evolutionary history of *P. aeruginosa* ST-1047 since its emergence in the late 19th century. Two predominant clones, one circulating in the United States and one rapidly emerging in Europe, have emerged via the gain of carbapenemase-harboring genomic islands. Both have shown outbreak potential. More subclones with distinct carbapenemases may be circulating in South Asia and the Middle East, but high-quality genomes are lacking. Sustained surveillance and high-resolution genomics are needed to capture the true prevalence, monitor the evolution, and mitigate the international spread of this lineage.

## Data Availability

Genomes sequenced by the MRSN have been deposited at GenBank under BioProjects PRJNA950451, PRJNA1162747, and PRJNA1281664. Accession numbers for all genomes are provided in Table S1.

## SUPPLEMENTAL MATERIAL

Figure S1 mbio.02020-25-s0001.pdf Species-level genetic analysis reveals ST-1047 emerged from a common ancestor with lineage ST-377.

Figure S2 mbio.02020-25-s0002.pdf Independent deletion of PrrF2 in IMP-carrying subclone 2.

Table S1 mbio.02020-25-s0003.xlsx Isolates, genomes, and AMRg.

Table S2 mbio.02020-25-s0004.xlsx SNP matrix.

ASM does not own the copyrights to Supplemental Material that may be linked to, or accessed through, an article. The authors have granted ASM a non-exclusive, world-wide license to publish the Supplemental Material files. Please contact the corresponding author directly for reuse.
