# Supplementary material for: Recurring acquisition of carbapenemase genes and global emergence of Pseudomonas aeruginosa ST-1047, a lineage shaped by geopolitical conflicts
Source: mBio. 2025 Oct 8;16(11):e02020-25. doi: 10.1128/mbio.02020-25 (PMC12607901; doi:10.1128/mbio.02020-25)
Supplement: Figure S1 — Species-level genetic analysis reveals ST-1047 emerged from a common ancestor with lineage ST-377. [file mbio.02020-25-s0001.pdf]

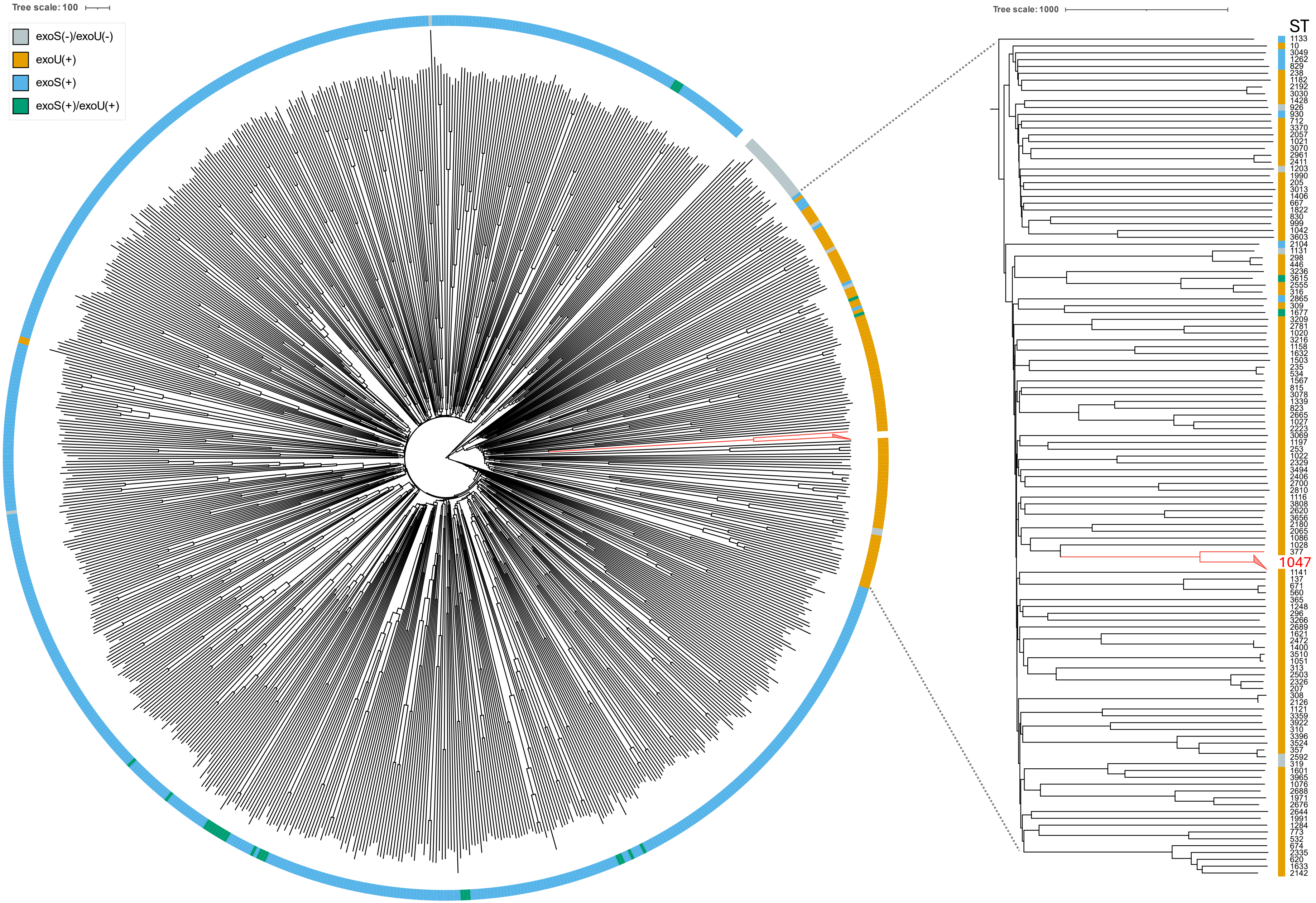

Supplemental Figure 1: Species-level genetic analysis reveals ST-1047 emerged from a common ancestor with lineage ST-377. Neighbor joining tree showing the genetic relatedness (based on a cgMLST distance matrix) of 43 ST-1047 isolates compared to 812 isolates representing the diversity of MLST sequence-types in the entire MRSN collection of *P. aeruginosa* genomes. Closest STs to ST-1047 are indicated as well as the presence of the *exoU*/*exoS* alleles.
