# Supplementary material for: Recurring acquisition of carbapenemase genes and global emergence of Pseudomonas aeruginosa ST-1047, a lineage shaped by geopolitical conflicts
Source: mBio. 2025 Oct 8;16(11):e02020-25. doi: 10.1128/mbio.02020-25 (PMC12607901; doi:10.1128/mbio.02020-25)
Supplement: Figure S2 — Independent deletion of PrrF2 in IMP-carrying subclone 2. [file mbio.02020-25-s0002.pdf]

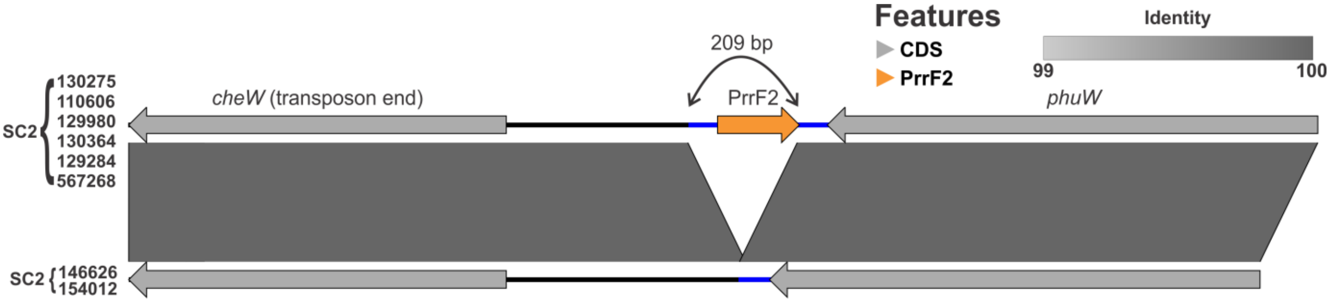

Supplemental Figure 2: Independent deletion of PrrF2 in IMP-carrying subclone 2. The PAGI-97B-like genomic island juncture with the chromosome in configuration 1 versus that of configuration 2. Configuration 2 has a 209 bp sequence containing PrrF2 excised. PrrF1 is located on the other end of the transposon, proximal to recombinase *xerD*.
